# TFE3‐PD‐L1 axis is pivotal for sunitinib resistance in clear cell renal cell carcinoma

**DOI:** 10.1111/jcmm.16066

**Published:** 2020-11-03

**Authors:** Xudong Guo, Ruxia Li, Qiulei Bai, Shaobo Jiang, Hanbo Wang

**Affiliations:** ^1^ Department of Urology Shandong Provincial Hospital Affiliated to Shandong First Medical University Jinan China

**Keywords:** clear cell renal cell carcinoma, PD‐L1, sunitinib, TFE3

## Abstract

The microphthalmia of bHLH‐LZ transcription factor (MiT/TFE) family chromosomal translocation or overexpression is linked with a poor prognosis in clear cell renal cell carcinoma (ccRCC) with elevated recurrence and drug resistance, but the molecular mechanism is not fully understood. Here, we investigated whether the resistance to sunitinib (Sun), the standard treatment for metastatic ccRCC, is due to up‐regulation of programmed death ligand 1 (PD‐L1) by the transcription factor E3 (TFE3). In this study, we propose that TFE3 but not TFEB is essential for tumour survival which was associated with the poorer survival of cancer patients. We also found a positive correlation between TFE3 and PD‐L1 expression in ccRCC cells and tissues. Sun treatment led to enhanced TFE3 nuclear translocation and PD‐L1 expression. Finally, we observed the therapeutic benefit of Sun plus PD‐L1 inhibition which enhanced CD8+ cytolytic activity and thus tumour suppression in a xenografted mouse model. These data revealed that TFE3 is a potent tumour promoting gene and it mediates resistance to Sun by induction of PD‐L1 in ccRCC. Our data provide a strong rationale to apply Sun and PD‐L1 inhibition jointly as a novel immunotherapeutic approach for ccRCC treatment.

## 1 INTRODUCTION

Renal cell carcinoma (RCC) has become the most malignant tumour of kidneys in worldwide.^1^ Clear cell renal cell carcinoma (ccRCC) is the most common subtype of RCCs, representing approximately 70% of all adult renal carcinomas. The other histologies mainly encompass papillary and chromophobe RCC.^2^ The prognosis of ccRCC is poor: 30% of patients are metastatic at diagnosis and almost 30% of the remaining patients will develop metastasis detected during the follow‐up.^3^ With the continuous development of medicine, the treatment and management of ccRCC, particularly metastatic RCC, have radically changed over the past few decades.^4^ Initially, first‐generation immunotherapy with cytokines: interleukins or interferon represented standard approaches but with poor results.^5^, ^6^ In recent years, the development of tyrosine kinase inhibitors (TKI), mainly targeted to vascular endothelial growth factor receptor, largely improved the prognosis of both overall survival (OS) and progression free survival (PFS).^7^ Sunitinib malate (Sun) is an oral multi‐targeting TKI that is registered for the treatment of advanced or metastatic RCC.^8^, ^9^ Currently, the emergence of immune checkpoint inhibitors (ICI) showed promising results in RCC treatment.^10^, ^11^, ^12^ Programmed cell death 1 (PD‐1), programmed death ligand‐1 (PD‐L1) and Cytotoxic T‐lymphocyte‐associated protein‐4 (CTLA‐4) could inhibit the proliferation and differentiation of immunocompetent cells and the recognition of tumour cells by tumour‐infiltrating lymphocytes (TILs). Currently, PD‐1/PD‐L1 axis has attracted massive interest.^13^ Blockade the PD‐1/PD‐L1 axis has been of benefit in the treatment of many different types of cancers including ccRCC.^14^, ^15^

TKI or ICI for the treatment of RCC has significantly improved the OS, PFS and durable responses in some patients. However, resistance and relapse are common; and only 15%‐25% of patients exhibit clinical responses to checkpoint blocking when given as monotherapy.^16^, ^17^ So, innovative combinations of TKI with ICI are now part of the treatment strategy and have achieved exciting benefits according to the results of recently updated phase III trials.^10^, ^18^, ^19^ However, the molecular mechanisms of these novel combinations need further investigations.

The MiT‐TFE family of basic helix‐loop‐helix leucine‐zipper transcription factors including TFEB, TFE3, TFEC and MITF play a major role as regulators of lysosome biogenesis, cellular energy homeostasis and immune responses; thus, they were originally described as oncogenes.^20^ The expression of the TFEB and TFE3 and their activity are elevated in multiple types of human cancers and associated with enhanced proliferation and motility of these cancer cells.^21^ Furthermore, TFEB or TFE3 fusion and overexpression caused by chromosomal translocation events is linked with a poor prognosis in a subset of RCC patients with elevated recurrence and metastasis.^22^ But the molecular mechanism is not fully understood. Recently, Zhang et al^23^ reported that TFEB mediates immune evasion and resistance to mTOR inhibition of RCC via induction of PD‐L1. These studies have shown that the MiT‐TFE family plays an important role not only in the progression, but also chemotherapy resistance of RCC tumours.

In this study, we found that TFE3 is also a potent tumour promotor just like TFEB in ccRCC. Importantly, TFE3 but not TFEB is essential for the survival of tumour cells. TFE3 can also regulate PD‐L1 expression in ccRCC cell lines and primary human ccRCC tumour tissues. We also found that Sun enhanced TFE3 nuclear translocation and PD‐L1 expression. Combination of Sun with anti‐PD‐L1 enhanced the therapeutic efficacy in a mouse RCC xenograft model. Thus, our data provide rationale for the combined use of Sun and PD‐L1 blockade as a potential therapeutic strategy to treat ccRCC.

## 2 MATERIALS AND METHODS

### 2.1 Cell culture and reagents

786‐O, A498, TK‐10 ccRCC cells, Renca mouse RCC cell and HepG2 liver adenocarcinoma cell (the Cell Bank of the Chinese Academy of Sciences) were cultured in RPMI‐1640 medium supplemented with 10% foetal bovine serum (HyClone) and 100 U/mL penicillin and 100 g/mL streptomycin. All these cells were routinely cultured in 5% CO_2_ at 37°C. After chemical treatments, cells were collected for Western blots or other assays. Sunitinib malate (#S1042) was purchased from Selleck. Anti‐mouse PD‐L1 antibody (BP0101) was purchased from BioXCell.

### 2.2 Western blots analysis and antibody

Cells or tumour tissues were washed with ice‐cold PBS and lysed in RIPA lysis buffer containing a fresh protease and phosphate inhibitor mixture (50 mg/mL aprotinin, 0.5 mmol/L phenylmethanesulfonyl fluoride, 1 mmol/L sodium orthovanadate, 10 mmol/L sodium fluoride and 10 mmol/L β‐glycerolphosphate). Cell lysates were then prepared for Western blots. Protein concentrations were quantified by BCA protein assay. Cell lysates were mixed with 4× loading buffer and heated at 95°C for 5 minutes. Equal volumes of lysates were run on 5%‐15% SDS‐PAGE gels and transferred to 0.2‐mm nitrocellulose membranes (GE, A29411350). Blots were blocked for 1 hour at room temperature in TBS with 0.05% Tween 20 (Sigma Aldrich, P7949) (TBS‐T) and 5% non‐fat milk. Primary antibodies were incubated overnight at 4°C in TBS‐T with 5% non‐fat milk. HRP‐conjugated secondary antibodies were incubated 1 hour at room temperature. Blots were washed with TBS‐T, three times, 5 minutes and TBS, one time, 5 minutes each after both primary and secondary antibody incubations. Blots were developed with Western Lighting Plus‐ECL (Thermo, TK275827) and exposed in dark room. Blots were normalized to GAPDH or β‐Actin loading controls. Blots were incubated with primary antibodies against GAPDH (Santa Cruz, Sc‐32233), Histone H3 (Cell Signaling Tech, 4499), β‐Actin (Santa Cruz, Sc‐47778), TFEB (Cell Signaling Tech, 37785), TFE3 (Cell Signaling Tech, 14779) and PD‐L1 (Abcam, ab213524) overnight at 4°C prior to being probed with the appropriate peroxide‐conjugated secondary antibodies.

### 2.3 Real‐time quantitative PCR

Total RNAs was extracted using an RNAiso plus kit (TaKaRa). Complementary DNA was synthesized through reverse transcription using ReverTra Ace qPCR RT Kit (TOYOBO). Quantitative PCR analysis of cDNA was performed with SYBRGreen reaction master mix on a Real‐time PCR System (Eppendorf International). Target mRNA levels were normalized to the level obtained for GAPDH. Changes in transcript level were calculated using DD△Ct method. The primers used in this experiment were listed in Table 1.

**Table 1:** Primer

Name (Human)	Forward primer	Reverse primer
*TFE3*	CCGTGTTCGTGCTGTTGGA	GCTCGTAGAAGCTGTCAGGAT
*TFEB*	CCAGAAGCGAGAGCTCACAGAT	TGTGATTGTCTTTCTTCTGCCG
*CD80*	TGCCTGACCTACTGCTTTGC	AGGGCGTACACTTTCCCTTC
*CD86*	CGACGTTTCCATCAGCTTGTC	CGCGTCTTGTCAGTTTCCAG
*CD273*	ACCAGTGTTCTGCGCCTAA	CCTGGGTTCCATCTGACTTTG
*CD274*	GGTAAGACCACCACCACCAAT	TGATTCTCAGTGTGCTGGTCAC
*CD275*	CGTCTTCTTGAACATGCGGG	TTTTCTCGCCGGTACTGACT
*CD276*	CTCACAGGAAGATGCTGCGT	CTGTGAGGCAGAACCACAGT
*VTCN1*	TCTGGGCATCCCAAGTTGAC	TCCGCCTTTTGATCTCCGATT
*VISTA*	ACGCCGTATTCCCTGTATGTC	TTGTAGAAGGTCACATCGTGC
*CD155*	AGGCTATAATTGGAGCACGACC	GGTTTGTCCACAGGACGGAT
*CD270*	CAAGGTGATCGTCTCCGTCC	TCTGTGGGTCAGTGGTTTGG
*GAL3*	ATAACCTGCCTTTGCCTGGG	AGCAATTCTGTTTGCATTGGGC
*HMGB1*	TATGGCAAAAGCGGACAAGG	CTTCGCAACATCACCAATGGA
*CD70*	GTCACTTGGGTGGGACGTA	CAGTATAGCCTGGGGTCCTG
*CD154*	ACATACAACCAAACTTCTCCCCG	GCAAAAAGTGCTGACCCAATCA
*CD252*	GAGCCCCTCTTCCAACTGAA	CAGTTCTCCGCCATTCACAT
*β‐ACTIN*	CATGTACGTTGCTATCCAGGC	CTCCTTAATGTCACGCACGAT
*GAPDH*	GGAGCGAGATCCCTCCAAAAT	GCTGTTGTCATACTTCTCATG

### 2.4 xCELLigence

Experiments were carried out using the RTCADP instrument (Roche) which was placed in a humidified incubator maintained at 37°C with 5% CO_2_. For time‐dependent cell response profiling, 10 000 cells/well were added to 16‐well E‐Plates. The electronic sensors provided a continuous and quantitative measurement of cell index in each well. Cell index is a quantitative measure of cell number present in a well, that is lower cell index reflects fewer cells are attached to the electrodes. The E‐Plate 16 was monitored over the time frame indicated.

### 2.5 ethynyl‐2'‐deoxyuridine (EdU) incorporation assay

EdU cell proliferation kit (17‐10527) was purchased from Millipore. Pretreatment with siRNA, the cells were incubated 16 hours at 37°C in complete media supplemented with 10 μmol/L EdU. After washing in PBS, the cells were fixed and permeabilized. Reaction cocktail and DAPI (Beyotime) were then added. The fluorescence change of cells was detected with flow cytometry or microscope.

### 2.6 Subcellular fractionation

Cells were lysed in NP‐40 lysis buffer containing 20 mmol/L Tris‐HCL (pH 7.9), 150 mmol/L NaCl, 0.5 mmol/L EDTA and 0.5% NP‐40 supplemented with protease and phosphatase inhibitors. Lysed cells were kept on ice for 15 minutes. The lysates were then centrifuged at 2000 *g* for 5 minutes. The resulting supernatants represented the cytosolic and membrane fractions. The corresponding pellets representing the nuclear fractions were washed one time in NP‐40‐containing lysis buffer and sonicated in nuclear lysis buffer (20 mmol/L Tris‐HCl [pH 7.4], 450 mmol/L NaCl, 0.5 mmol/L EDTA, 0.5% Triton X‐100, 0.1% SDS). The lysates were then centrifuged at 12 000 *g* for 15 minutes to obtain the cytosolic and nuclear fractions.

### 2.7 Microscopy

To measure TFE3 nuclear translocation, cells following TFE3‐GFP transfection and Sun (5 µmol/L) treatments were incubated with DAPI for 10 minutes. The cells were then washed with PBS, and nuclear translocation fluorescence was measured using confocal microscopy (Carl Zeiss).

### 2.8 Patient samples

Clear cell RCC and benign samples were obtained from surgical excision specimens at the Shandong Provincial Hospital. Utilization of the clinical samples was approved by the Ethical Committee of the Shandong Provincial Hospital affiliated to Shandong First Medical University.

### 2.9 Immunohistochemistry (IHC)

Heat‐induced epitope retrieval was performed in 10 mmol/L citric acid buffer (pH 7.2) using a microwave. The slides were incubated at 4°C overnight with primary antibodies (anti‐PD‐L1, 1:200 dilution; anti‐TFE3, 1:200 dilution). An HRP‐conjugated antibody and 3,30‐diaminobenzidine (DAB) staining were used to visualize primary antibody binding. High‐resolution pictures were obtained on a digital electron microscope, and images were recorded using Case Viewer software. Immunohistochemical results are expressed as a mean score that considers both the intensity of the staining and a positive reaction.

### 2.10 Transfection

Cells were transfected with specifically targeted TFEB (AGACGAAGGUUCAACAUCA), TFE3 (CGCAGGCGATTCAACATTAAC) and the plasmid of TFE3‐GFP, using Lipofectamine 2000 Transfection Reagent.

### 2.11 Flow cytometry

The PD‐L1 expression in the cells was determined using flow cytometry. Cells following various treatments were collected by centrifugation. After two washes with ice‐cold PBS, cells were stained with antibodies against PE‐conjugated anti‐human PD‐L1 antibody. The antibodies used to stain TILs were listed as followed: anti‐CD3‐FITC (100203; Biolegend), anti‐CD4‐APC (100516; Biolegend), anti‐CD8‐Percp/Cy5.5 (100734; Biolegend) and anti‐GZMB‐PE (104508; Biolegend).

### 2.12 Luciferase reporter assay

Luciferase reporter assay was identified as reported previously^23^ and modified slightly. The *PD‐L1* promoter sequence (−250 to +45 bp) was amplified by PCR from human 786‐0 cell and inserted into the pGL3‐basic vector (pGL3‐PD‐L1). 293T cell were co‐transfected with pRL‐TK, pGL3‐basic or pGL3‐PD‐L1, pcDNA3.1, TFEB or TFE3 plasmids in 12‐well plates. Then, renilla luciferase activities were measured using the Dual‐Luciferase Reporter Assay Kit (E1901; Promega) with microplate reader (CYT5M; BioTek).

### 2.13 Xenograft mouse tumour models

C57BL/6 mice (6 weeks old) were obtained from the Animal Center of the China Academy of Medical Sciences. Murine RCC cell Ruca were injected into the right flanks of the mice and allowed to establish tumours. When the tumours reached 50‐100 mm^3^, the mice were given the clinical chemotherapeutics sunitinib (40 mg/kg, i.p.) daily, anti‐PD‐L1 (200 μg/mouse, i.p.). Tumour volumes (mm^3^) were calculated from the formula 0.5 × L×W^2^ (L = length, W = width). All animal experiments were approved by the Ethics Committee of the Shandong University School of Medicine.

### 2.14 Statistical analysis

Western blots and fluorescent images were analysed with Image Pro Plus 6.0. The data are presented as the mean ± SD and were analysed with GraphPad Prism software (GraphPad). Student's *t* test or one‐way ANOVA was used for comparisons among different groups. Kaplan‐Meier and Cox proportional hazards analyses were used for survival analysis. All the experiments were repeated at least three times. Values of *P* < .05 denoted statistical significance and are indicated as *P ≤ .05, **P ≤ .01 and ***P ≤ .001 in the figures.

## 3 RESULTS

### 3.1 TFE3 but not TFEB affects cell proliferation of ccRCC cells

**Figure 1:**
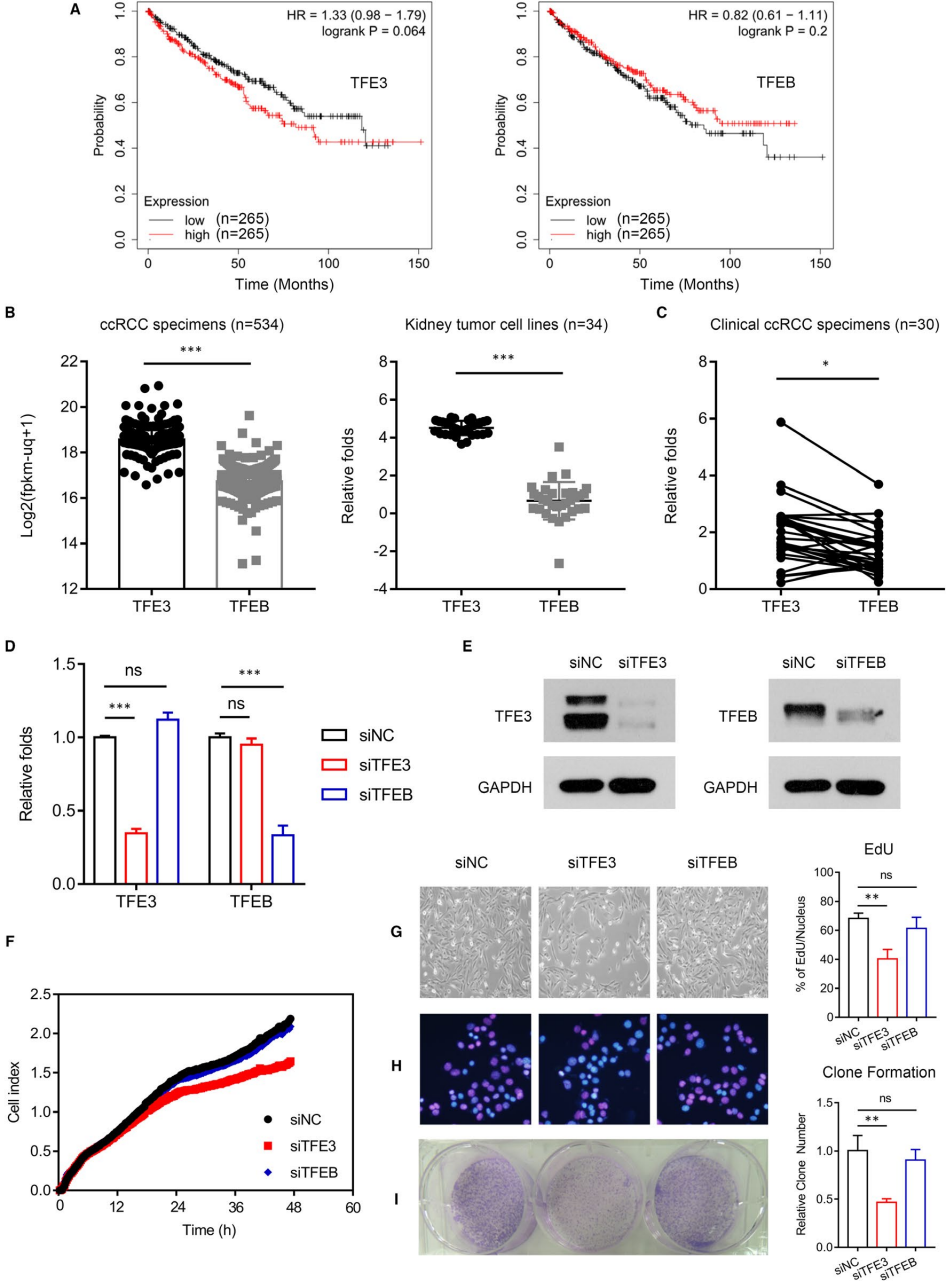
TFE3 but not TFEB affect cell proliferation of ccRCC cells. A, The relationship between TFE3/TFEB and patient prognosis in ccRCC was analysed in data from Kaplan‐Meier plotter database. B, The expression of TFE3 and TFEB in ccRCC specimens and RCC cells were analysed in data from TCGA and CCLE database. C, The expression of TFE3 and TFEB in ccRCC specimens was analysed by qPCR. D, siRNA knockdown of TFE3 and TFEB was analysed by qPCR. E, siRNA knockdown of TFE3 and TFEB was detected by Western blots. F, Cell viability was analysed using a xCELLigence RTCADP instrument. G, siRNA knockdown of TFE3 and TFEB was performed, and the cell proliferation was analysed by morphology. H, siRNA knockdown of TFE3 and TFEB was performed, and the cell proliferation was analysed by EdU. I, siRNA knockdown of TFE3 and TFEB was performed, and the cell proliferation was analysed by clone formation. Data are mean ± SD, *P < .05, **P < .01 and ***P < .001

The levels of TFEB and TFE3 are elevated in multiple types of human cancers and have been linked with both occurrence and poor prognosis.^20^, ^21^, ^22^, ^24^ Recent study claimed that TFEB has little effect on RCC proliferation.^23^ To clarify the role of TFEB and TFE3 in ccRCC, we first analysed the publicly available Kaplan‐Meier plotter and The Cancer Genome Atlas (TCGA) database on the expression of TFEB and TFE3. As shown in Figure 1A, TFE3 but not TFEB was negatively correlated with the survival of patients in clear cell RCC. We also found that the basal expression of TFE3 is higher than that of TFEB in ccRCC specimens and tumour cell lines by TCGA and Cancer Cell Line Encyclopedia (CCLE) database (Figure 1B). To further validate the database information, we examined 30 clinical renal clear cell carcinoma samples by qPCR and confirmed that TFE3 expression is higher (Figure 1C). To determine whether this difference defines the proliferative advantage of ccRCC, we tried to knockdown TFEB and TFE3 in 786‐O cells (Figure 1D,E). As a result, knockdown of TFEB did not affect cell proliferation. This is consistent with previous report.^23^ But interestingly, knockdown of TFE3 significantly inhibited cell proliferation (Figure 1F). These results were further validated by EdU staining and clone formation assay (Figure 1G‐I). In addition, we also knocked down TFEB and TFE3 in hepatocellular carcinoma cells and got similar results (Figure S1A‐D). This indicated that TFE3 can be at least another potent tumour promotor beyond TFEB in specific tumour types such as ccRCC.

### 3.2 TFE3 mediates immune evasion by positively regulation the expression of PD‐L1 in ccRCC cells and ccRCC patients

**Figure 2:**
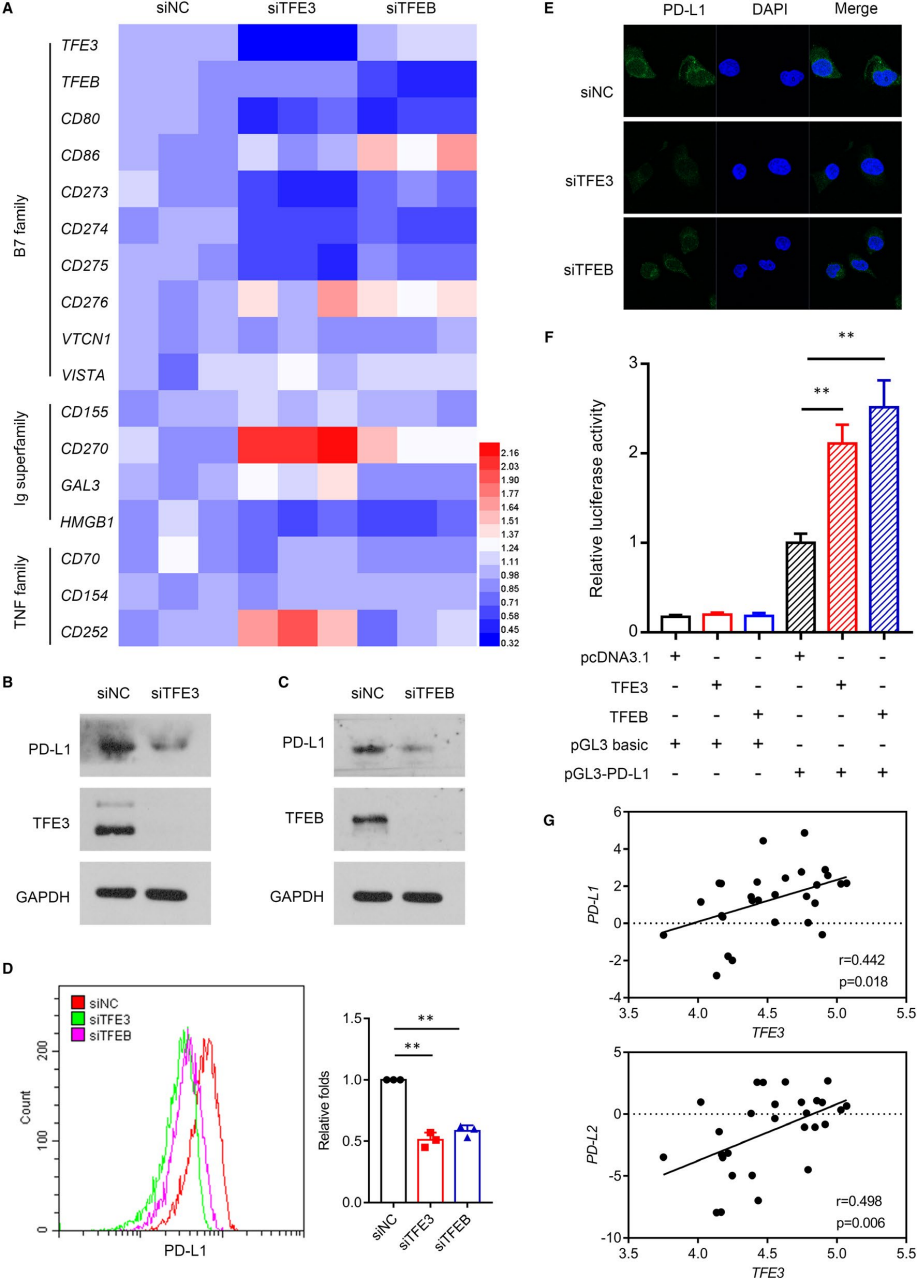
TFE3 mediates immune evasion by positively regulation the expression of PD‐L1 in ccRCC cells and ccRCC patients. A, siRNA knockdown of TFE3 and TFEB was performed in 786‐O cell, and the heat map of immune checkpoints‐related gene expression was analysed by qPCR. B, TFE3 was knocked down with siRNA, and the expression of PD‐L1 was detected by Western blots in 786‐O cell. C, TFEB was knocked down with siRNA, and the expression of PD‐L1 was detected by Western blots in 786‐O cell. D, TFE3 and TFEB were knocked down with siRNA, and the expression of PD‐L1 was detected by flow cytometry. E, TFE3 and TFEB were knocked down with siRNA, and the expression of PD‐L1 was detected by immunofluorescent in 786‐O cell. F, 293T cells were transfected with the pGL3 basic plasmids, or pGL3‐PD‐L1‐Luc plasmids together with pcDNA3.1 or TFEB or TFE3 plasmids for 48 h, then luciferase activities were determined. G, The correlation between *TFE3* and *PD‐L1* and *PD‐L2* was analysed by CCLE database. Data are mean ± SD, **P < .01

Recent study showed that TFEB can mediate immune evasion by positively regulation the expression of PD‐L1 in RCC.^23^ Encouraged by the similar roles of TFE3 and TFEB in the regulation of cell fate, we next explored whether TFE3 also can mediate immune evasion by regulating the expression of immune checkpoint markers, such as PD‐L1. As shown in Figure 2A, TFE3 and TFEB can both regulate the expression of some immune checkpoint markers, such as CD273 (PD‐L2), CD274 (PD‐L1) and CD275 (ICOSL). To further clarify the regulation of TFE3 on the expression of PD‐L1, we first down‐regulated TFE3 and TFEB in multiple cells (A498, TK‐10, HepG2) and then found that PD‐L1 was down‐regulated accordingly (Figure S2A). These results were further confirmed by Western blots, immunofluorescence and flow cytometry (Figure 2B‐E). Furthermore, TFE3 overexpression can significantly enhance luciferase activity driven by the *PD‐L1* promoter (Figure 2F). We also found that expression of *TFE3* but not *TFEB* was positively correlated with the levels of *PD‐L1* in RCC tumour cell lines by CCLE database (Figure 2G and S2B). Then, we choose TFE3 for further study.

**Figure 3:**
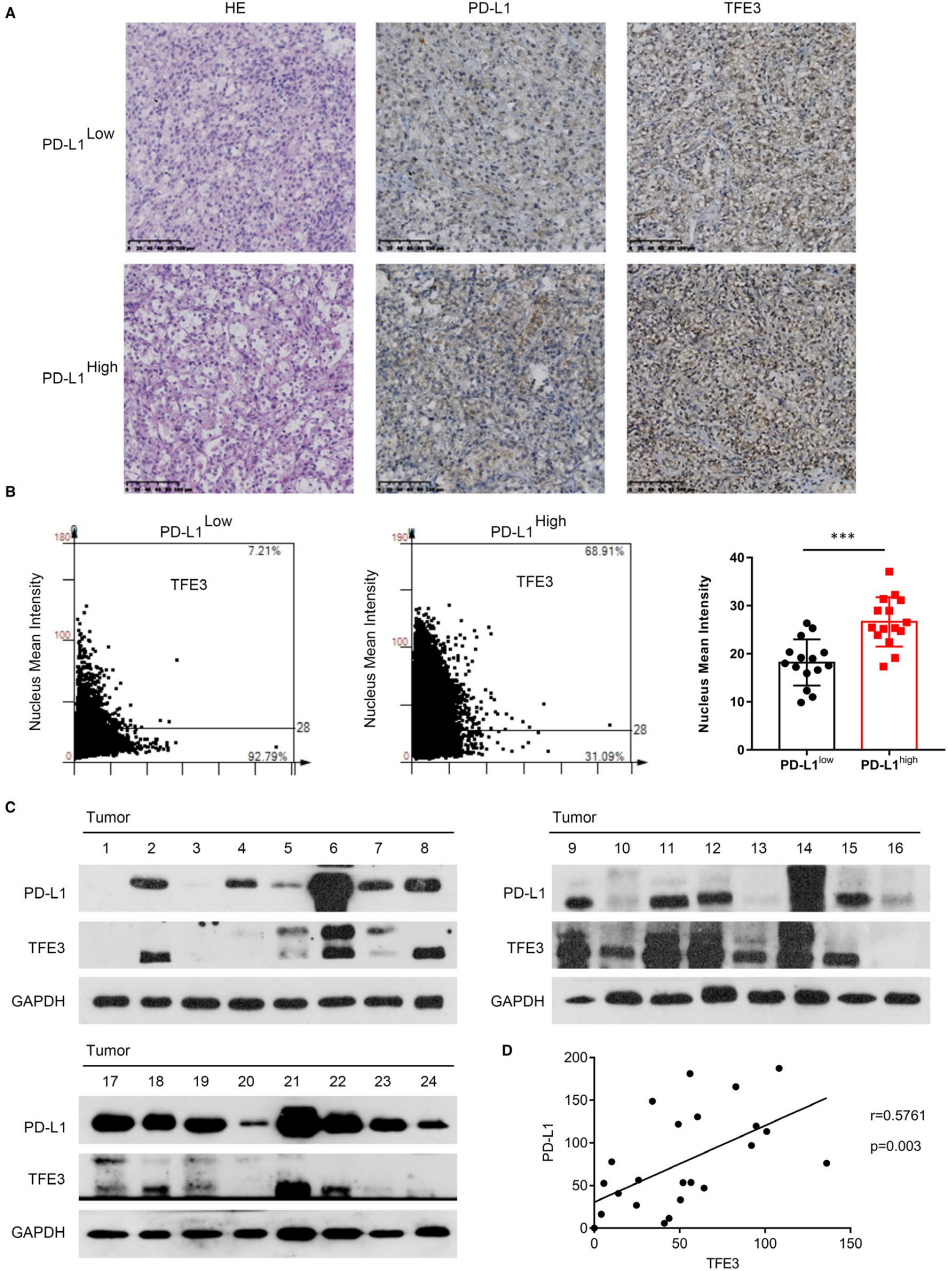
TFE3 mediates immune evasion by positively regulation the expression of PD‐L1 in ccRCC cells and ccRCC patients. A, H&E and IHC staining with TFE3 and PD‐L1 on human ccRCC tissues. B, The nucleus mean intensity of TFE3 in human PD‐L1‐negative and PD‐L1‐positive ccRCC tissues measured by IHC (n = 30). C, TFE3 and PD‐L1 expression in ccRCC tissues were determined by Western blots (n = 24). D, The correlation of TFE3 and PD‐L1 expression in ccRCC tissues was plotted (n = 24). Data are mean ± SD, ***P < .001

We next evaluated whether the level of TFE3 correlated with PD‐L1 expression in primary ccRCC patients. Within individual tumours, PD‐L1 staining showed heterogeneous expression, which can be readily differentiated into PD‐L1^−^ and PD‐L1^+^ areas. Higher expression and enhanced nuclear localizations of TFE3 were seen in the PD‐L1^+^ regions (Figure 3A,B). These results were further confirmed by Western blots (Figure 3C,D). Together, these findings demonstrate that TFE3 can positively regulate PD‐L1 expression.

### 3.3 Sunitinib enhances PD‐L1 expression via activation of TFE3 in ccRCC cells

**Figure 4:**
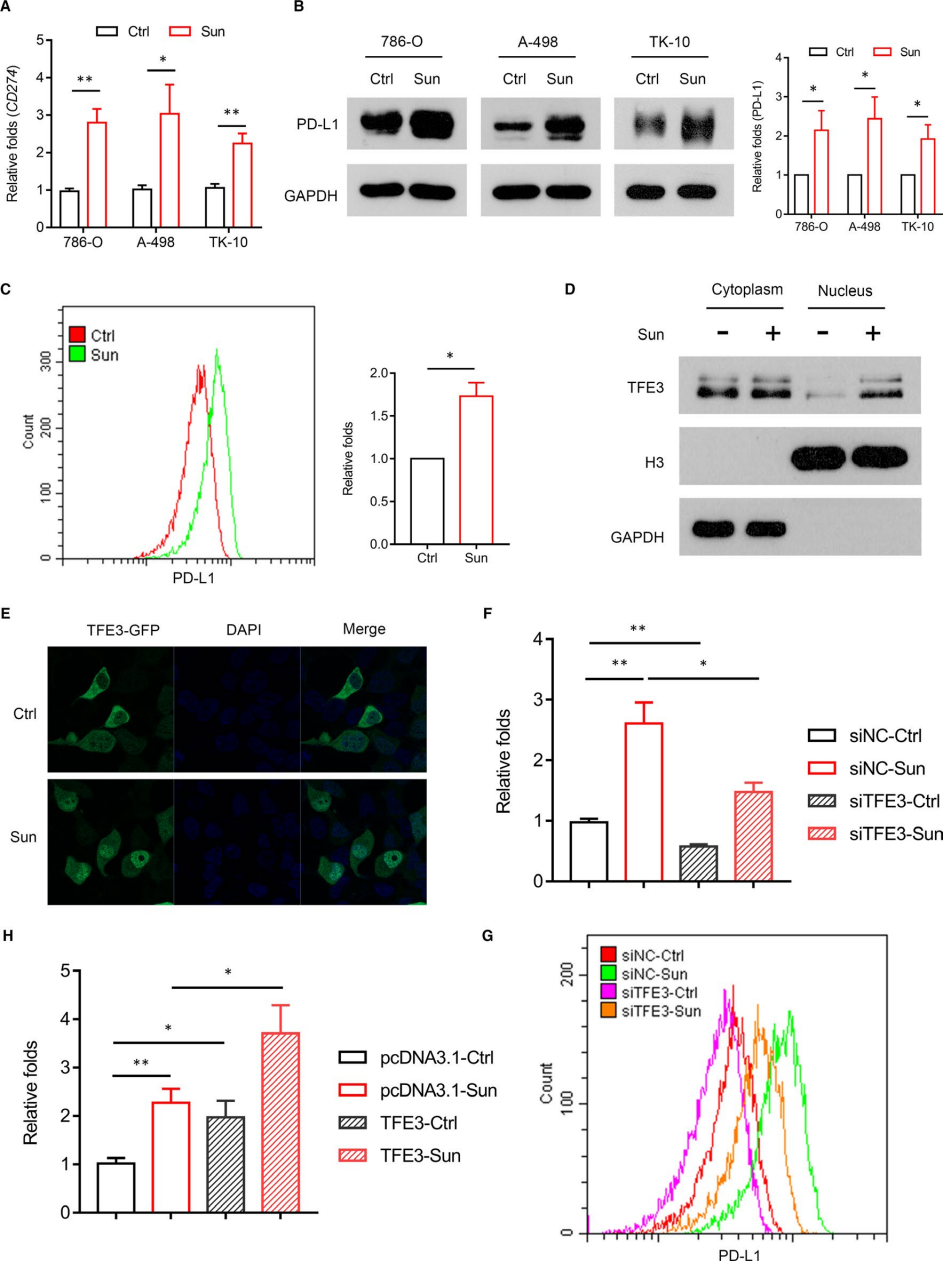
Sunitinib enhances PD‐L1 expression via activation of TFE3 in ccRCC cells. A, *PD‐L1* expression was analysed by qPCR in multiple cells (786‐O, A498, TK‐10) with sunitinib treatment. B, PD‐L1 expression was analysed by Western blots in multiple cells (786‐O, A498, TK‐10) with sunitinib treatment. C, PD‐L1 expression was analysed by flow cytometry in 786‐O cell with sunitinib treatment. D, Western blot analysis of the nuclear translocation of TFE3 in 786‐O cell with sunitinib treatment. E, Immunofluorescent staining showing the TFE3 state in nuclear and cytosolic fractions of 786‐O cell incubated with sunitinib. F, siRNA knockdown of TFE3 was performed in combination with sunitinib treatment, and the expression of *PD‐L1* was analysed by qPCR. G, siRNA knockdown of TFE3 was performed in combination with sunitinib treatment, and the expression of PD‐L1 was analysed by flow cytometry. H, Cells overexpressing TFE3 were treated with a combination of sunitinib, and the expression of *PD‐L1* was analysed by qPCR. Data are mean ± SD, *P < .05 and **P < .01

Sunitinib is an oral TKI that is currently registered for the treatment of advanced or metastatic RCC.^8^, ^9^ Despite its initial excitement for the treatment of ccRCC, Sun rarely achieved complete responses and most patients ultimately developed resistance to Sun therapy, and the mechanism of resistance is not fully understood yet. Recent study reported that Sun increased PD‐L1 expression in liver tumour cells.^25^ Therefore, we proposed that the tolerance induced by Sun can also be related to the up‐regulation of PD‐L1 expression in ccRCC cells. As shown in Figure 4A,B, Sun can induce the expression of PD‐L1 in different ccRCC cells (786‐O, A‐498, TK‐10). These results were confirmed using flow cytometry of PD‐L1 in 786‐O cell (Figure 4C). Given that TFE3 can regulate the expression of PD‐L1 in our study, next we tried to see whether Sun enhanced PD‐L1 expression dependent of TFE3 expression. As shown in Figure 4D,E, Sun treatment significantly enhanced TFE3 nuclear accumulation in 786‐O cell. Knocking down TFE3 inhibited PD‐L1 expression, and Sun‐induced PD‐L1 levels were also noticeably decreased in cells lacking of TFE3 (Figure 4F,G). In contrast, the ectopic expression of TFE3 induced PD‐L1 expression in the presence of Sun (Figure 4H). Together, these data demonstrated that Sun can induce PD‐L1 expression by activation of TFE3 in human ccRCC cells.

### 3.4 Anti‐PD‐L1 immunotherapy enhances the response to sunitinib in RCC

**Figure 5:**
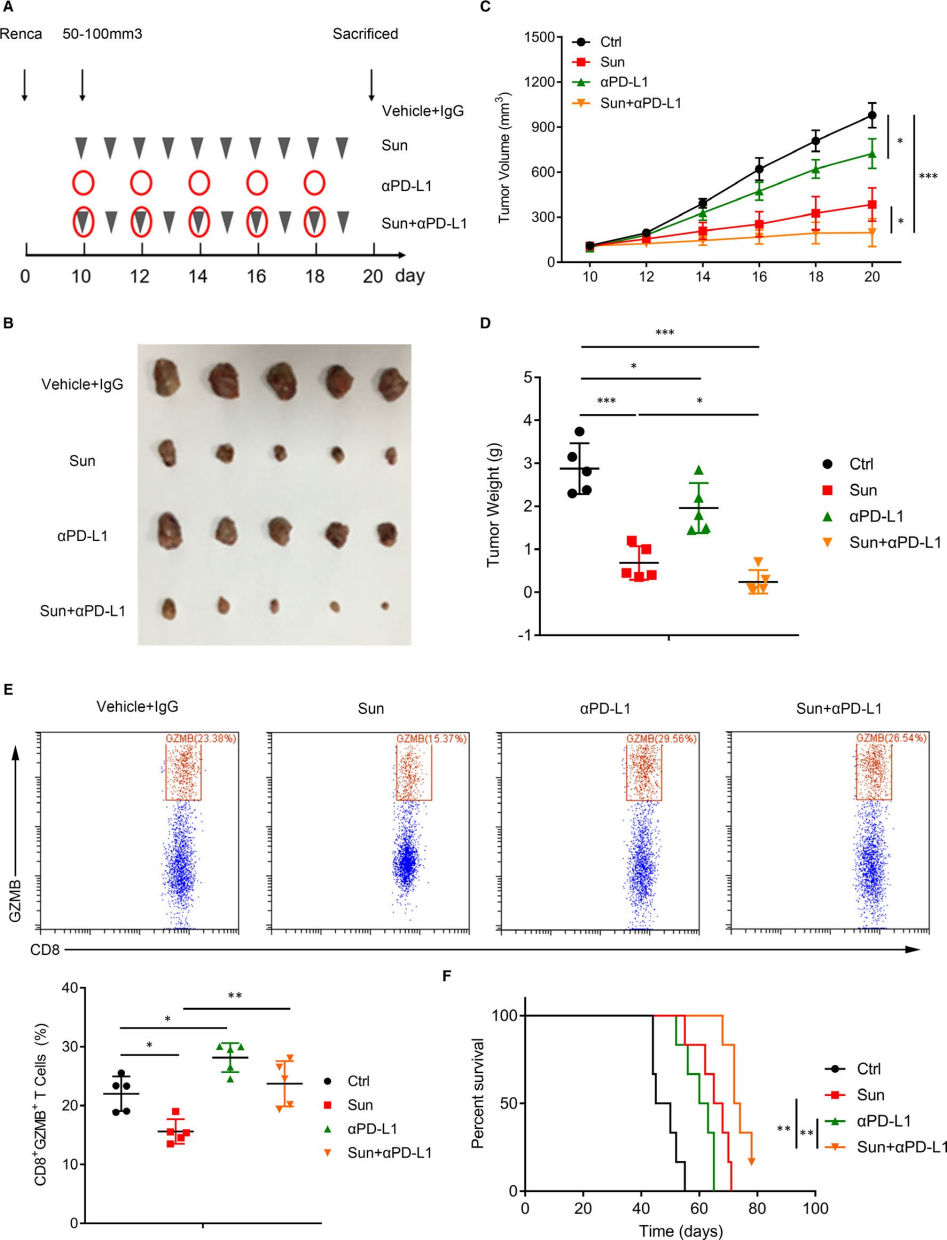
Anti‐PD‐L1 immunotherapy enhances the response to sunitinib in RCC. A, Model of the animal experiment. B, Photographs of excised tumours from four groups (vehicle and IgG, sunitinib [40 mg/kg], anti‐PD‐L1 [200 μg/mouse], or a combination of sunitinib and anti‐PD‐L1) are shown. C, Tumour volumes in different groups were recorded every 2 d. D, Tumour weights from four groups are shown. E, TILs were isolated and stained with CD8 and GZMB. Representative histograms shown on the left panel. Percentages of CD8^+^ GZMB^+^ were shown on the right panel. F, Homograft mice model showed overall survival difference from four groups. Data are mean ± SD, *P < .05, **P < .01 and ***P < .001

We then asked whether combined use of PD‐L1 antibody could potentiate the efficacy of Sun on ccRCC growth in the xenograft mouse model. When tumour volume reached 50 mm^3^, mice were treated with either sunitinib (40 mg/kg, i.p.) daily, anti‐PD‐L1 (200 μg/mouse, i.p.) five times over 12 days, combination of both or vehicle plus control IgG (Figure 5A). There was a reduction in tumour growth in mice treated with either anti‐PD‐L1 alone or Sun alone compared with the control group (Figure 5B‐D). By contrast, the combination of Sun and anti‐PD‐L1 therapy resulted in a significant reduction in tumour size compared with all the other groups (Figure 5B‐D). Next, we tested the effect of Sun and PD‐L1 inhibition on cytotoxicity in tumour‐infiltrating CD8^+^ T cells (CTL). Sun treatment suppressed GZMB expression in CTL, but when combined with anti‐PD‐L1, Sun significantly enhanced their expression (Figure 5E). The combination treatment also resulted in increased survival (Figure 5F). Together, these data demonstrated that the combined use of Sun and anti‐PD‐L1 can be a novel immunotherapeutic approach for ccRCC treatment.

## 4 DISCUSSION

Numerous studies have provided evidence suggesting that MiT/TFE transcription factors are important for the maintenance of cellular physiological and pathological processes.^24^ Among all the four members of the MiT/TFE family, TFEB and TFE3 show a more ubiquitous pattern of expression and their functions has been widely investigated including proliferation, metabolism and autophagy.^20^, ^24^ The tight connection of TFEB and TFE3 with RCC has been reported, especially in translocation renal cell carcinoma.^21^, ^22^, ^23^ But its biological function is not clearly investigated. We have found that the expression of TFE3 is higher than TFEB in tumour cells and patients. More interestingly, TFE3 but not TFEB has intrinsic effects on cell proliferation and survival in ccRCC and LIHC. This was consistent with the patient's prognosis. We also knocked down TFE3 and TFEB in lung cancer and breast cancer cells where their expression has no negative correlation with patient prognosis and it was found that alteration of the TFE3 or TFEB expression has little effect on tumour maintenance or progression (data not shown). These results suggest that TFE3 and TFEB are different in the regulation of biological processes, except for controlling autophagic and/or lysosomal function. And their specific molecular mechanisms need further study.

Identification of the TFE3‐regulated genes in ccRCC cells can provide better understanding of TFE3 functions in the regulation ccRCC tumorigenesis and the interaction between ccRCC cells and the immune microenvironment. Recent study showed that TFEB can mediate immune evasion by positively regulation the expression of PD‐L1 in RCC.^23^ Given the similar roles of TFE3 and TFEB in the cross‐regulation of cellullar functions, we tested the expression of typical immune checkpoints, and interestingly, we found that TFE3 can also regulate the expression of many immune checkpoints including CD273 (PD‐L2), CD274 (PD‐L1), CD275 (ICOSL) and CD270 (HVEM). Our study revealed a strong correlation between PD‐L1 protein level and TFE3 expression in ccRCC cells and ccRCC patients (Figures 2 and 3). Although, Zhang, et al reported that the expression of TFE3 was not associated with PD‐L1 expression in RCC cell lines. This may be as a result of the limited number of RCC cell lines (five cells) which they used in they study. Using the publicly available Broad Institute CCLE database, we found that there is a positive correlation between *TFE3* and *PD‐L1* (*P* = .018), *PD‐L2* (*P* = .006), expression in 28 human RCC cell lines. So, TFE3 and TFEB cooperate in the regulation of the immune evasion in ccRCC.

Sunitinib is an oral TKI that is currently registered for the treatment of advanced or metastatic RCC, gastrointestinal stromal tumour and neuroendocrine tumour.^8^, ^26^, ^27^ Despite the early success of Sun on the treatment for ccRCC, most patients ultimately developed resistance whose mechanism is not fully understood. In fact, the resistance has been largely attributed to the derailing of intracellular signalling pathways, but less on the immune microenviromment.^8^, ^9^ In this study, we demonstrated that Sun led to enhanced nucleus translocation of TFE3 in RCC cells, which subsequently induces PD‐L1 expression. Furthermore, combination of Sun and anti‐PD‐L1 enhanced the cytotoxic functions of tumour‐infiltrating CTL and therapeutic efficacy in a mouse RCC xenograft model.

In summary, it is indicated that TFE3, like TFEB, is also a potent tumour promotor based on its significant proliferative effect. And more importantly, it mediates PD‐L1 up‐regulation, which can ultimately attenuate Sun therapeutic efficacy via tumour‐associated immune‐suppression. By emphasizing on the pivotal role of TFE3, our data provide a valuable rational for the application of chemoimmunotherapy on the RCC patients.

## CONFLICT OF INTEREST

None.

## AUTHOR CONTRIBUTIONS

**Hanbo Wang:** Conceptualization (equal); Data curation (supporting); Funding acquisition (lead); Project administration (lead); Resources (lead); Supervision (equal); Validation (equal); Visualization (equal); Writing‐original draft (lead); Writing‐review & editing (lead). **Xudong Guo:** Conceptualization (equal); Data curation (lead); Formal analysis (lead); Methodology (lead); Project administration (supporting); Writing‐original draft (supporting); Writing‐review & editing (supporting). **Ruxia Li:** Data curation (supporting); Formal analysis (supporting); Investigation (supporting); Software (equal). **Qiulei Bai:** Data curation (supporting); Formal analysis (supporting); Methodology (supporting); Software (equal). **Shaobo Jiang:** Project administration (equal); Supervision (equal); Validation (equal).

## Supporting information

Fig S1‐2

## Data Availability

The data sets used and/or analysed during the current study are available from the corresponding author on reasonable request.
