# Supplementary material for: TFE3‐PD‐L1 axis is pivotal for sunitinib resistance in clear cell renal cell carcinoma
Source: J Cell Mol Med. 2020 Nov 3;24(24):14441–52. doi: 10.1111/jcmm.16066 (PMC7753981; doi:10.1111/jcmm.16066)

**TFE3-PD-L1 axis is pivotal for sunitinib resistance in** **clear cell renal cell carcinoma**

**Running title:** TFE3-PD-L1 axis is pivotal for sunitinib resistance

Xudong Guo^1^, Ruxia Li^1^, Qiulei Bai^1^, Shaobo Jiang^1^, Hanbo Wang^1*^

**Supplementary Figure Legends**

Figure 1. **TFE3 but not TFEB affect cell proliferation of ccRCC cells**

(A) The relationship between TFE3/TFEB and patient prognosis in Liver hepatocellular carcinoma (LIHC) was analyzed in data from Kaplan Meier plotter database. (B) The expression of TFE3 and TFEB in LIHC specimens and LIHC cells were analyzed in data from TCGA and CCLE database. (C) siRNA knockdown of TFE3 and TFEB was analyzed by qPCR. (D) [Cell](javascript:;) [viability](javascript:;) was analyzed using a xCELLigence RTCADP instrument. Data are mean ± SD, *P < 0.05, **P < 0.01 and ***P < 0.001.

Figure 2 **TFE3 mediates** **immune evasion by positively regulation the expression of PD-L1 in ccRCC cells and cc****RCC patients**

(A) The expression of *PD-L1* was analyzed by qPCR in multiple cells (A498, TK-10, HepG2). (B) The correlation between *TFEB* and *PD-L1*, *PD-L2* were analyzed by CCLE database. Data are mean ± SD, *P < 0.05, **P < 0.01 and ***P < 0.001.

**Supplementary** Figure 1:


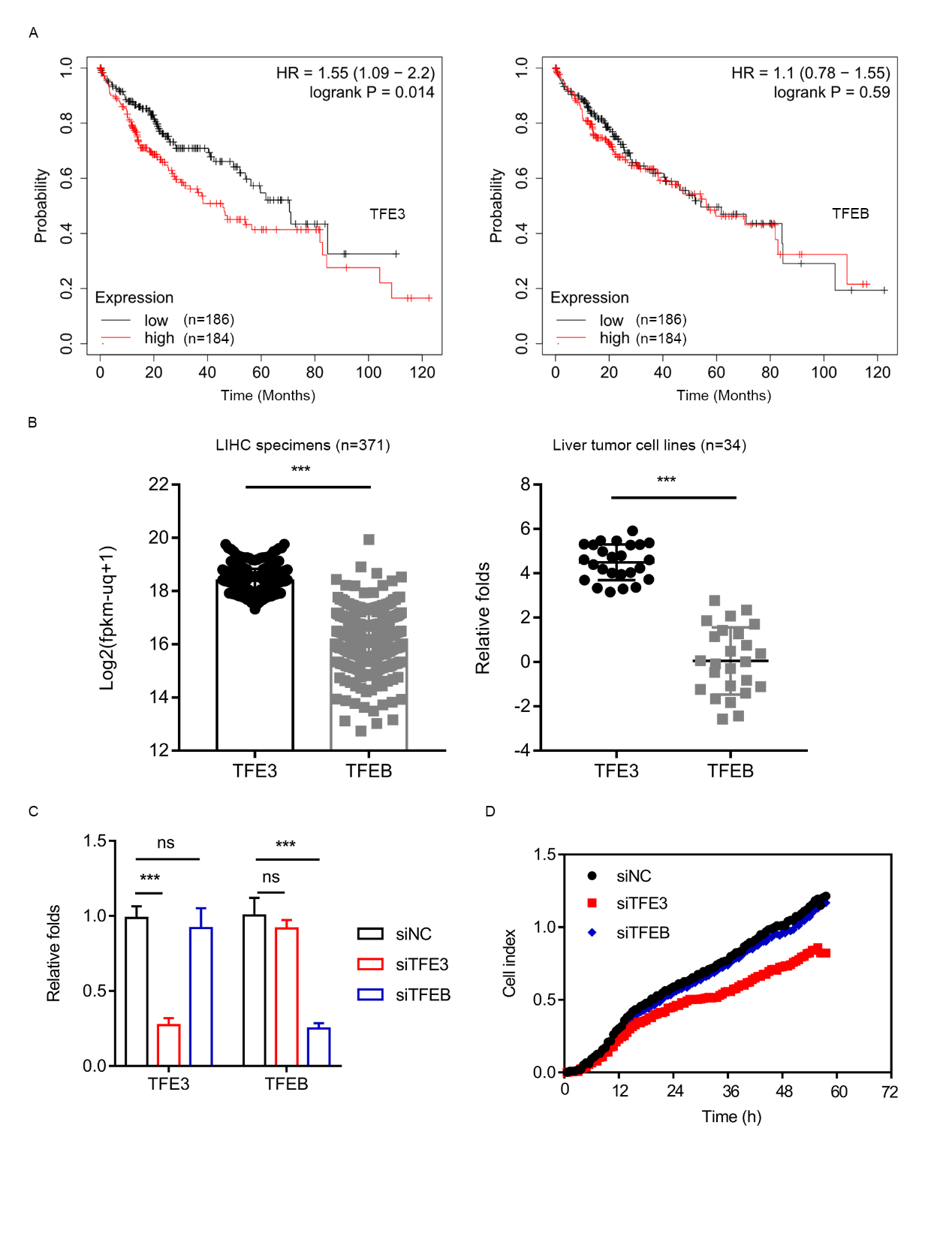


**Supplementary** Figure 2:


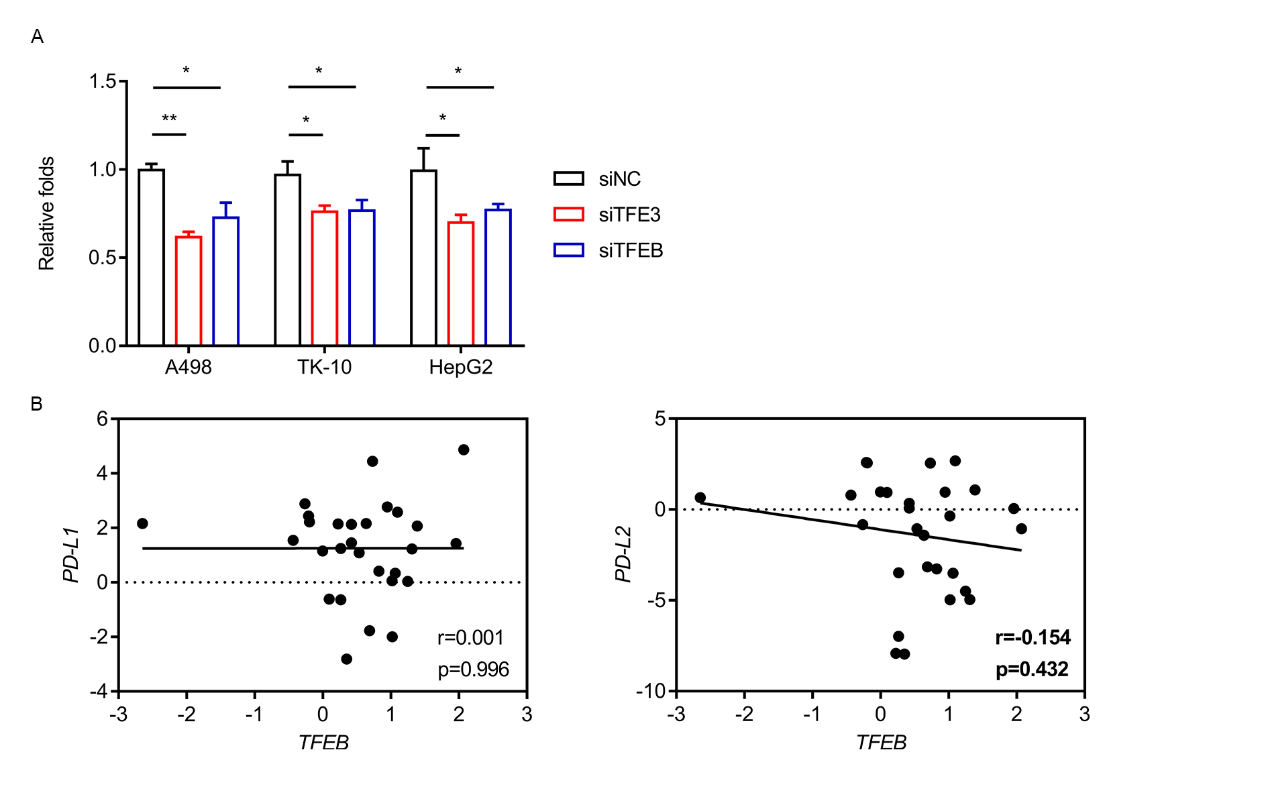

Supplement: Supplementary file 1 — Fig S1‐2 [file JCMM-24-14441-s001.docx]
